# Deeper Insights on *Cnesmone javanica* Blume Leaves Extract: Chemical Profiles, Biological Attributes, Network Pharmacology and Molecular Docking

**DOI:** 10.3390/plants10040728

**Published:** 2021-04-08

**Authors:** Ahmad J. Obaidullah, Mohammed M. Alanazi, Nawaf A. Alsaif, Wael A. Mahdi, Omer I. Fantoukh, Abu Montakim Tareq, Saad Ahmed Sami, Ali M. Alqahtani, Talha Bin Emran

**Affiliations:** 1 Department of Pharmaceutical Chemistry, College of Pharmacy, King Saud University, P.O. Box 2457, Riyadh 11451, Saudi Arabia; mmalanazi@ksu.edu.sa (M.M.A.); nalsaif@ksu.edu.sa (N.A.A.); 2 Department of Pharmaceutics, College of Pharmacy, King Saud University, P.O. Box 2457, Riyadh 11451, Saudi Arabia; wmahdi@ksu.edu.sa; 3 Department of Pharmacognosy, College of Pharmacy, King Saud University, P.O. Box 2457, Riyadh 11451, Saudi Arabia; ofantoukh@ksu.edu.sa; 4 Department of Pharmacy, International Islamic University Chittagong, Chittagong 4318, Bangladesh; montakim0.abu@gmail.com; 5 Department of Pharmacy, Faculty of Biological Sciences, University of Chittagong, Chittagong 4331, Bangladesh; s.a.sami18pharm@gmail.com; 6 Department of Pharmacology, College of Pharmacy, King Khalid University, Abha 62529, Saudi Arabia; amsfr@kku.edu.sa; 7 Department of Pharmacy, BGC Trust University Bangladesh, Chittagong 4381, Bangladesh

**Keywords:** *Cnesmone javanica*, anxiolytic, antidepressant, GC–MS, molecular docking, network pharmacology

## Abstract

This study assessed the anxiolytic and antidepressant activities of a methanol leaves extract of *Cnesmone javanica* (CV) in Swiss albino mice. The study found a significant increase in the percentage of time spent in the open arms of an elevated plus maze and in the incidence of head dipping in hole-board tests following the administration of 400 mg/kg of CV or 1 mg/kg diazepam. Moreover, a significant (*p* < 0.001) dose-dependent reduction was observed in the immobility time following CV (200 and 400 mg/kg) and fluoxetine (20 mg/kg) administration for forced swimming and tail suspension tests. Gas chromatography–mass spectroscopy (GC–MS) analysis identified 62 compounds in CV, consisting primarily of phenols, terpenoids, esters, and other organic compounds. A molecular docking study was performed to assess the anxiolytic and antidepressant effects of 45 selected compounds against human serotonin transporter and potassium channels receptors. Network pharmacology was performed to predict the pathways involved in these neuropharmacological effects. Overall, CV demonstrated significant and dose-dependent anxiolytic and antidepressant effects due to the presence of several bioactive phytoconstituents, which should be further explored using more advanced and in-depth mechanistic research.

## 1. Introduction

Medical plant use has achieved growing interest in developing countries, representing first therapeutic approach in 80% of developing countries. A significant proportion of the world’s population (87.5%) uses herbal remedies to treat health problems [1]. Many plants’ medicinal properties, their effects on the human body, and their treatment methods have been known since the 18th century, but their active compounds were unknown [2].

Mental disorders represent a large public health threat, affecting nearly 450 million people globally, and representing 12.3% of all diseases worldwide [3,4]. Mental disorders are among the world’s most alarming health problems, and the two most common mental health disorders are depression and anxiety [5,6]. If significant actions are not taken on a global level, the health problems associated with mental disorders are expected to become increasingly serious. Several gaps and inconsistencies exist in the current understanding of the numerous issues associated with mental health disorders, and treatment resources are often insufficient, particularly in low-income and developing countries [6]. Although several anxiolytic and antidepressant drugs are used clinically for the treatment of these disorders, currently available drugs are associated with several undesirable side effects [6,7,8,9]. Therefore, effective medications with fewer side effects are urgently necessary for the treatment of mental disorders.

Recently, researchers have begun to focus on the development of novel natural product remedies from local products, particularly plants, which have improved efficacy and reduced toxicity compared to currently available treatment options [6]. The literature has described several herbal mechanisms for the treatment of depression, anxiety, and insomnia [10].

*Cnesmone javanica* belongs to the Euphorbiaceae family, known as Lampinak [11,12]. The leaves of the *C. javanica* plant are traditionally eaten as vegetables. The root is often used in a juice form to treat fever and can also be used as a poisonous substance [12]. The cytotoxic, larvicidal, and repellent effects of *C. javanica* have been reported using different components and extraction methods [12]. However, no additional effects or chemical compounds have been investigated.

The present study explored the anxiolytic and antidepressant activities of a methanol extract of *C. javanica* (CV), combined with a gas chromatography–mass spectroscopy (GC–MS) analysis. A molecular docking study was also used to understand the compounds’ molecular interactions with the anxiolytic and antidepressant receptors.

## 2. Results and Discussion

**Figure 1:**
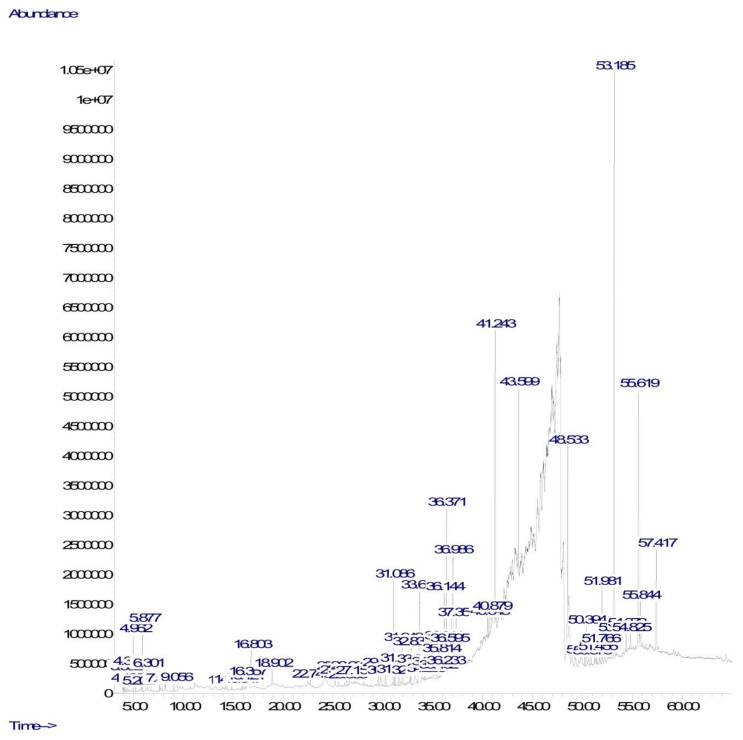
Total ionic chromatogram (TIC) of methanol extract of *Cnesmone javanica* (CV) using gas chromatography–mass spectrometry (GC–MS).

The present study identified several phytoconstituents in CV. The potential therapeutic effects associated with *C. javanica* could be associated with the presence of these bioactive phytoconstituents. The analysis was conducted using GC–MS, which represents one of the most commonly used methods for the separation of phytoconstituents. The GC–MS-based investigation of CV identified 62 phytochemical compounds (Table 1). Figure 1 illustrates a typical chromatogram for CV. The major phytoconstituents included phenols, terpenoids, phytosterols, esters, and other organic compounds. The major compounds included dl-α-tocopherol (23.53% and 3.72%), 9,12,15-octadecatrienoic acid (10.44%), β-sitosterol (9.07%), hexanedioic acid, bis(2-ethylhexyl) ester (7.89%), squalene (6.31%), n-hexadecanoic acid (1.17% and 5.77%), phthalic acid, 2-ethylhexyl tetradecyl ester (5.23%), and phytol (4.35%). Tocopherol derivatives are reported to exert neuroprotective effects and are most commonly available in the form of vitamin E [13,14]. The phenolic compounds have also been reported to possess neurological effects [15].

### 2.1. Effects of CV on Anxiolytic and Antidepressant Activity

Rodent exploratory behavior has recently stimulated the interest of researchers in several behavioral pharmacology studies [16]. The behavioral tests used in this study are known to measure anxiolytic and antidepressant activities, and most have been previously validated [17,18]. The EPM and HBT represent the two most commonly used methods for screening anxiolytic effects [19]. The elevated plus maze (EPM) exposes the animal to elevated open and closed arms, and time spend in the open arm is an indication of reduced anxiety [18,20]. In behavioral pharmacology, the hole-board test (HBT) is a common test of anxiolytic activity, in which the increased frequency of head dipping through the holes is considered an indication of reduced anxiety behavior in mice [16,21]. The tail suspension test (TST) and forced swimming test (FST) are the most commonly used methods to assess antidepressant-like activity in rodents [22]. The immobility behavior expressed by mice during these tests is considered to represent a depressive state [22,23]. Animal immobility induced by the FST and TST have been reported to model human depression and can be reversed with the application of antidepressant drugs [23].

**Figure 2:**
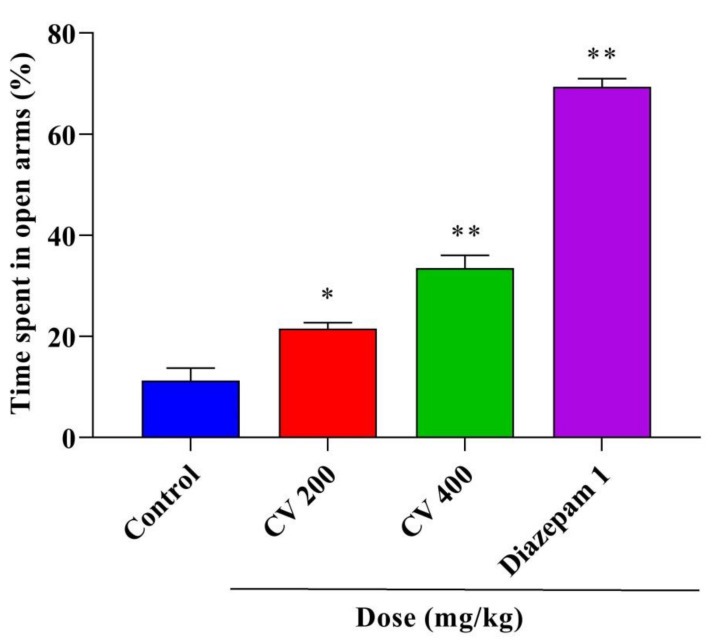
Anxiolytic activities of the methanol extract of *Cnesmone javanica* (CV) and diazepam as assessed by the elevated plus maze test. Significant differences (* *p* < 0.01 and ** *p* < 0.001) were assessed using one-way analysis of variance (ANOVA, followed by Dunnett’s test).

**Figure 3:**
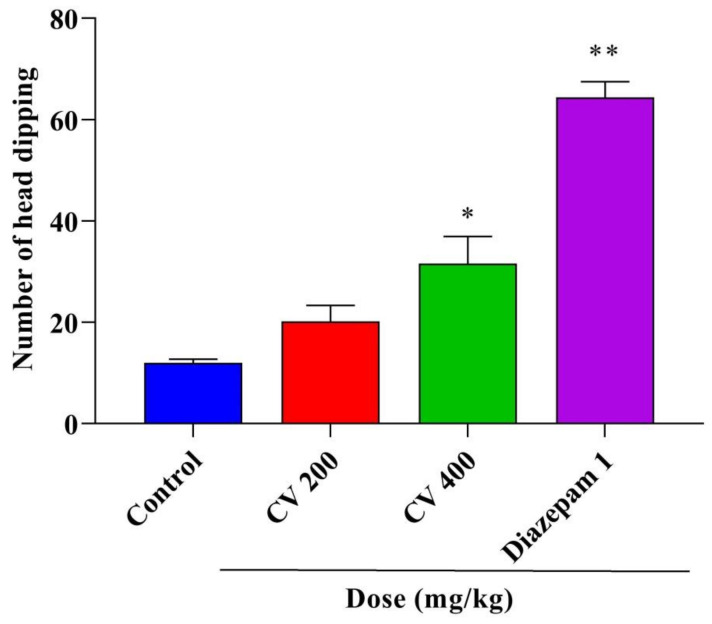
Anxiolytic activities of the methanol extract of *Cnesmone javanica* (CV) and diazepam as assessed by the hole-board test. Significant differences (* *p* < 0.01 and ** *p* < 0.001) were assessed using one-way analysis of variance (ANOVA, followed by Dunnett’s test).

The findings for the EPM, shown in Figure 2, demonstrated that the percentages of time spent in the open arms were 21.52% and 33.48% in mice treated with CV doses of 200 (*p* < 0.01) or 400 mg/kg (*p* < 0.001), respectively, which were significantly increased compared with that of the control group (11.25%). However, the intraperitoneal (IP) administration of the positive control diazepam (1 mg/kg) significantly (*p* < 0.001) increased the percentage of time spent in the open arm (69.33%). The results of the HBT, shown in Figure 3, indicated that the oral administration of CV at 400 mg/kg and the IP administration of 1 mg/kg diazepam significantly increased the frequency of head dipping (*p* < 0.01 and *p* < 0.001, respectively) compared with mice in the control group. However, no significant difference was identified between the control and 200 mg/kg CV treatment.

**Figure 4:**
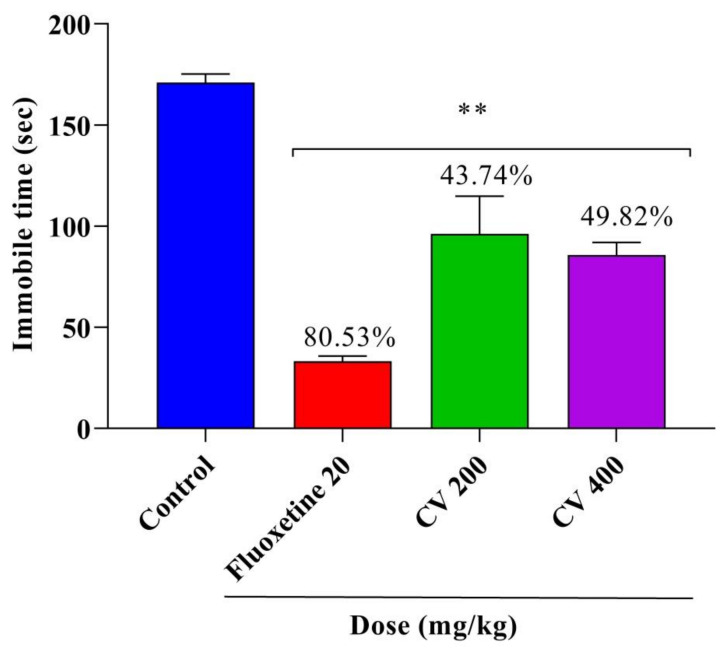
Antidepressant activities of the methanol extract of *Cnesmone javanica* (CV) and fluoxetine as assessed by the forced swim test. Significant differences (** *p* < 0.001) were assessed using one-way analysis of variance (ANOVA, followed by Dunnett’s test).

**Figure 5:**
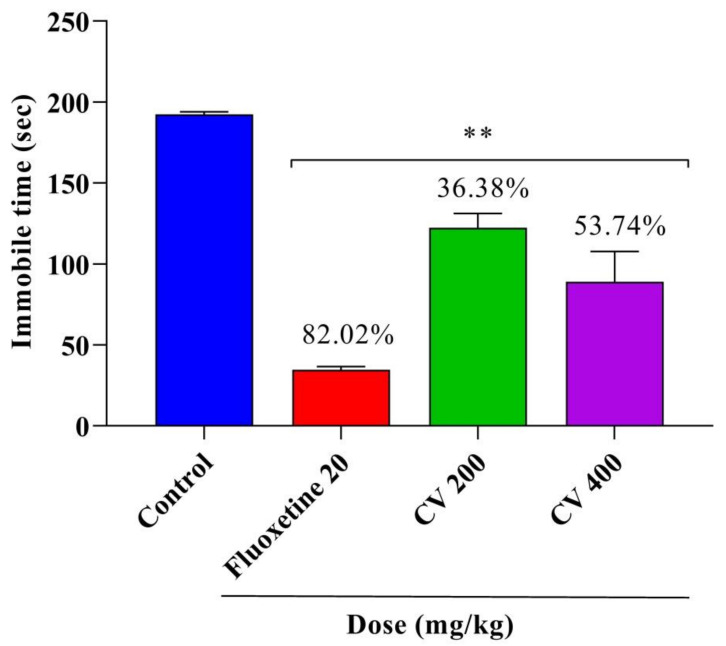
Antidepressant activities of the methanol extract of *Cnesmone javanica* (CV) and fluoxetine as assessed by the tail suspension test. Significant differences (** *p* < 0.001) were assessed using one-way analysis of variance (ANOVA, followed by Dunnett’s test).

The antidepressant effects of CV were assessed using the FST. As shown in Figure 4, the FST demonstrated that the immobility index of mice was reduced by 43.74%, 49.82%, and 80.53% at doses of 200 and 400 mg/kg CV and 20 mg/kg fluoxetine (*p* < 0.001), respectively, compared with that of the control group. A similar observation was found for the TST, as shown in Figure 5. Compared with the control group, CV (200 and 400 mg/kg) and fluoxetine (20 mg/kg) treatment significantly (*p* < 0.001) reduced the immobility index in the TST by 36.38%, 53.74%, and 82.02%, respectively.

Gamma-aminobutyric acid (GABA) is an inhibitory neurotransmitter (NT) found in the central nervous system (CNS) [24]. GABA receptor dysfunction has been associated with an increased risk of both anxiety and depression in both pharmacological and genetic studies [25]. Recent studies have reported that the inactivation of the γ-2 subunit gene, which results in a partial decrease in GABA_A_ receptor function in the mouse brain, resulted in an increased risk of anxiety and depressive behaviors in adults [24,26]. Similar to clinical outcomes in humans, mice with γ-2 subunit deficiencies showed reduced anxiety behaviors when treated with diazepam or with the chronic use of fluoxetine [24,26,27]. Furthermore, in antidepressant-sensitive tests, such as FST and TST, the γ-2 subunit-deficient mice also exhibited significantly improved depression-like responses [24,27,28,29]. These studies indicate that the GABAergic deficiency that has been reported among patients with anxiety and depression may represent a causative factor for both disorders. Therefore, targeting the GABA system can provide a novel avenue for therapeutic strategies to treat both disorders, such as the use of selective GABA_A_ receptor agonists [24]. In the tentative identification of phytoconstituents in CV, the GC–MS analysis revealed several compounds that could potentially act on the GABA system.

### 2.2. Molecular Docking

Molecular docking has become an excellent tool for the discovery of novel drugs based on structural molecular biology [30,31]. Molecular docking has proven to be a very efficient strategy for identifying small molecules that bind to protein targets, and the modeling of protein–ligand docking is often used by the pharmaceutical industry to screen potentially active compounds [32]. In our present study, the tentative compounds identified in CV were used for the molecular docking study, as presented in Table 2.

**Figure 6:**
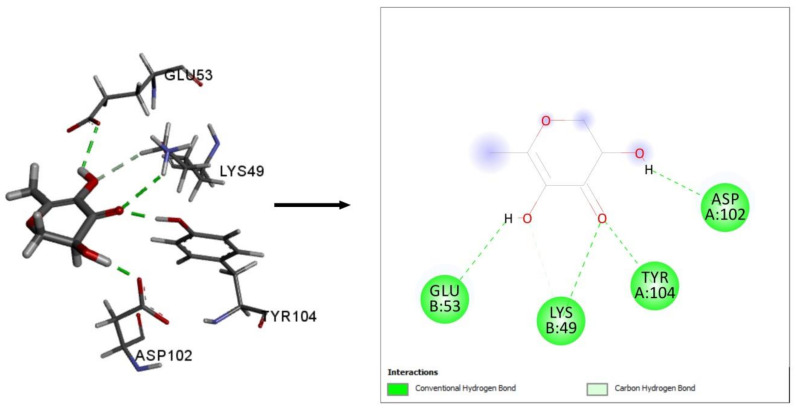
Best-docked interaction between 4H-Pyran-4-one, 2,3-dihydro-3,5-dihydroxy-6-methyl- and potassium channels receptor (PDB ID: 4UUJ).

To explore anxiolytic activity, a molecular docking study was used to examine the interactions between the compounds identified in CV and the potassium channels receptor (PDB ID: 4UUJ). Ion channels play a significant role in neuropsychiatric conditions, according to studies, and the potassium channel has potency against anxiety [33,34]. Interestingly, all of the phytochemicals identified in CV interacted with the potassium channels. Among these phytochemicals, 4H-pyran-4-one, 2,3-dihydro-3,5-dihydroxy-6-methyl- displayed the highest binding affinity (−4.887 kcal/mol), and the docking score approached that of the positive control, diazepam (−3.475 kcal/mol; Table 3 and Figure 6). The next highest docking scores were identified for 2-furancarboxaldehyde, 5-methyl- (−4.244 kcal/mol), furfural (−4.077 kcal/mol), DL-α-tocopherol (−4.041 kcal/mol), and 3-hydroxy-beta-damascone (−3.896 kcal/mol). 4H-pyran-4-one, 2,3-dihydro-3,5-dihydroxy-6-methyl-, 2-furancarboxaldehyde, 5-methyl-, furfural, 2,3,5,6-fetrafluoroanisole, and megastigmatrienone formed a hydrogen bond with the Lys49 and Tyr104 residue of the target receptor, while the positive control diazepam had similar interactions. Here, 2-furancarboxaldehyde, 5-methyl- interacted with Tyr50, Asp102 through the formation of hydrophobic bonds. Diazepam, which was used as the positive control in this study, formed hydrophobic interactions with Tyr50, Glu53, and Asp102 within the target receptor. Since tyrosine is a precursor for dopamine, noradrenaline, and epinephrine, these neurotransmitters are thought to be involved in fear suppression [35]. However, lysine also exhibited a reduction of anxiety in humans [36].

**Figure 7:**
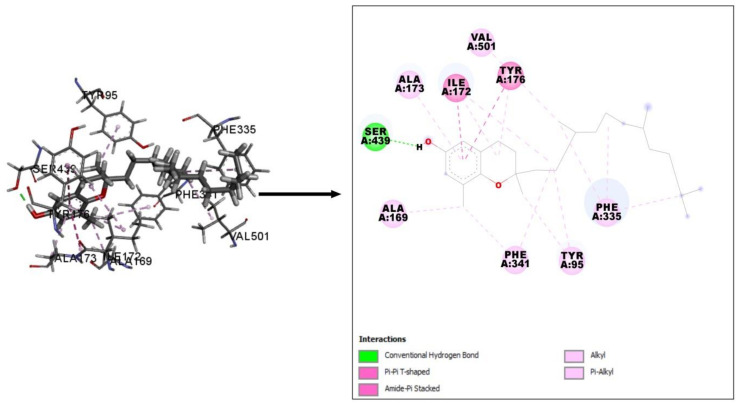
Best-docked interaction between 2H-1-benzopyran-6-ol, 3,4-dihydro-2,8-dimethyl-2-(4,8,12-trimethyltridecyl)-, [2R-[2R*(4R*,8R*)]]- and human serotonin receptor (PDB ID: 5I6X).

To examine antidepressant activity, a molecular docking simulation study was used to explore the interactions between selected compounds identified in CV and human serotonin receptor (PDB ID: 5I6X). The serotonin receptor has a significant dominance in depression, while 15 documented serotonin receptors were involved in depression-like behavior [37]. Interestingly, all of the phytochemicals identified in CV interacted with human serotonin receptor. Among these phytochemicals, some compounds possessed higher docking scores with the receptor than others. 2H-1-benzopyran-6-ol, 3,4-dihydro-2,8-dimethyl-2-(4,8,12-trimethyltridecyl)-, [2R-[2R*(4R*,8R*)]]- displayed the highest binding affinity (−9.303 kcal/mol), followed by γ-tocopherol (−8.384 kcal/mol), DL-α-tocopherol (−8.285 kcal/mol), β-tocopherol (−7.609 kcal/mol), β-sitosterol (−7.361 kcal/mol), and campesterol (−7.242 kcal/mol). 2H-1-benzopyran-6-ol, 3,4-dihydro-2,8-dimethyl-2-(4,8,12-trimethyltridecyl)-, [2R-[2R*(4R*,8R*)]]- interacted with our target receptor through the formation of a hydrogen bond with Ser439 and hydrophobic bonds with Tyr95, Ala169, Ile172, Ala173, Tyr176, Phe335, Phe341, and Val501. Both γ-tocopherol and DL-α-tocopherol demonstrated affinity toward the receptor through the formation of a hydrogen bond with Asn177 and hydrophobic interactions with Tyr95, Ala169, Ile172, Ala173, Tyr176, Phe335, Phe341, Gly442, Leu443, and Val501. 2H-1-benzopyran-6-ol, 3,4-dihydro-2,8-dimethyl-2-(4,8,12-trimethyltridecyl)-, [2R-[2R*(4R*,8R*)]]- exerted an even higher binding affinity than paroxetine (−8.978 kcal/mol), the positive control used in this study. Paroxetine interacted with the target receptor through the formation of hydrogen bonds with Tyr95 and Ala96, and hydrophobic bonds with Tyr95, Asp98, Ala169, Ile172, Ala173, Tyr176, Ser336, Phe341, Ser438, and Ser439 (Table 4 and Figure 7).

**Figure 8:**
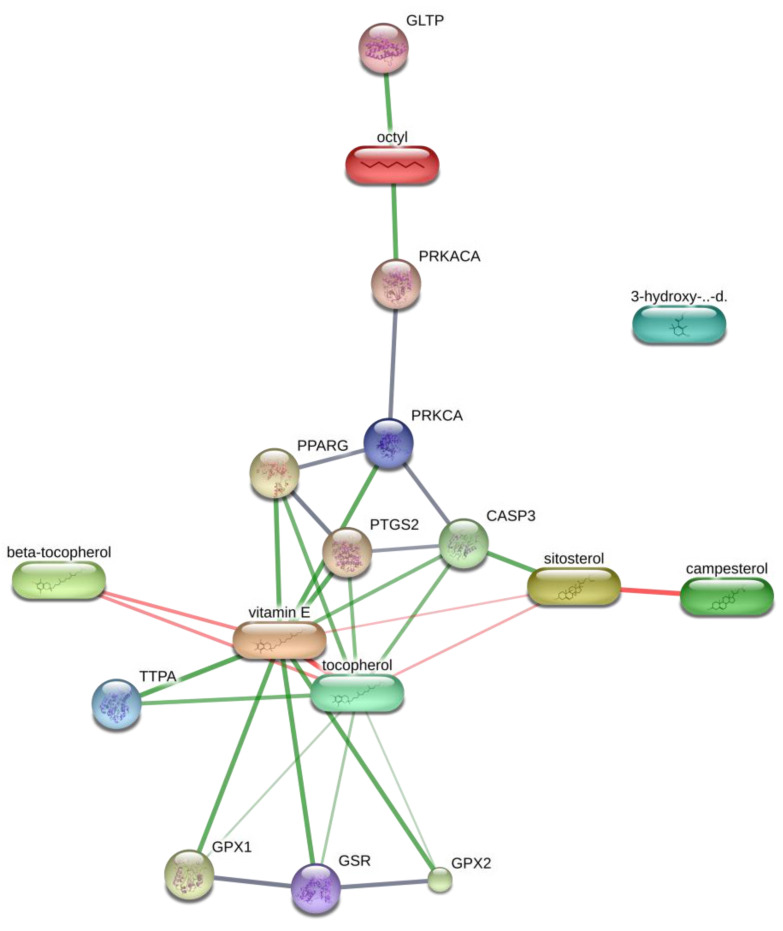
Network pharmacology presentation of phytoconstituents, targets, and pathways. Here, GLTP: Glycolipid transfer protein, PRKACA: Protein kinase CAMP-activated catalytic subunit alpha; PPARG: Peroxisome proliferator-activated receptor gamma; CASP3: Caspase 3; PTGS: Prostaglandin-endoperoxide synthase; TTPA: Alpha tocopherol transfer protein; GPx: Glutathione peroxidase; GSR: Glutathione-disulfide reductase; PRKCA: Protein kinase C-alpha.

Additionally, network pharmacology was used to explore the interactions among the 14 best-docked compounds using the STITCH platform. Interactions were identified for β-tocopherol, γ-tocopherol, DL-α-tocopherol (vitamin E), campesterol, and β-sitosterol with several genes. In the KEGG analysis, six different pathways were identified as being directly correlated with the neuropharmacology of CV (Table 5 and Figure 8), including the serotonergic synapse (*CASP3*, *PRKACA*, *PRKCA*, and *PTGS2*), GABAergic synapse (*PRKACA*, *PRKCA*), cholinergic synapse (*PRKACA*, *PRKCA*), glutamatergic synapse (*PRKACA*, *PRKCA*), dopaminergic synapse (*PRKACA*, *PRKCA*), Wnt signaling pathway (*PRKACA*, *PRKCA*), and Parkinson’s disease (*CASP3*, *PRKACA*). The biological process associated with the identified phytoconstituents is presented in Table 6, and the molecular functions are listed in Table 7. Future research remains necessary to analyze the compounds identified in the extract and to validate the existing results using individual biological models.

## 3. Materials and Methods

### 3.1. Preparation and Optimization of the Methanol Extract

The leaves of *C. javanica* were collected from the Lohagara Hill area in Chittagong, Bangladesh, in February 2020, which was identified by a renowned taxonomist from Bangladesh Forest Research Institute, Chittagong, Bangladesh. The leaves were air-dried and ground into a coarse powder. The crude extraction procedure was optimized using an established procedure [38]. Briefly, at a ratio of 1:4, the powder was macerated in methanol for seven days, after which the extract was filtrated by Whatman filter paper (#1), and the filtrate was evaporated at 40–45 °C, resulting in a black, semisolid extract. The CV extract was stored at 4 °C until use.

### 3.2. GC–MS Analysis

An Agilent GC 7890A (Agilent Technologies Inc., Wilmington, DE, USA), combined with a triple-axis detector 5975 C single-quadrupole mass spectrometer, was used for GC–MS analysis. The chromatographic column was an Agilent HP 5MS column (30 m × 0.25 mm × 0.25 µm film thickness), with high-purity helium as the gas carrier, at a flow rate of 1 mL per min. The injector temperature was 230 °C, and the sample was injected using a splitless injector at 20:1. The temperature was set primarily to 40 °C (held for 1 min), and then raised to 150 °C at a rate of 5 °C per min (held for 2 min), before being increased to 300 °C at a rate of 5 °C per min (held for 10 min). The temperature of the MS ion source was set to 150 °C, and the temperature of the inlet line was set to 280 °C. The scan range was set between 50–550 mass ranges, with 70 eV electron energy and a 4-min solvent delay. Finally, by comparing the spectra against the NIST 2008 database (National Institute of Standard and Technology library), tentative compounds were identified. The total analysis time required for the sample was 65 min.

### 3.3. Animals and Experimental Design

Swiss albino mice (20–25 g), six weeks old and of either sex, were randomly used for all tests. Mice were acclimated to the laboratory conditions for seven days, at a temperature of 25 ± 2 °C with free access to food and water. The institutional animal ethical committee approved this study, which followed the ARRIVE guidelines, in accordance with government regulations [39,40].

The mice were randomly distributed into four groups (*n* = 5) for each experiment. Two doses (200 and 400 mg/kg) of CV were used in this study, which were administered orally through gavage. Diazepam (1 mg/kg) was administered intraperitoneally (IP) for the elevated plus maze (EPM) and hole-board test (HBT). Fluoxetine was also administrated intraperitoneally for the forced swimming test (FST) and tail suspension test (TST). As a negative control, 1% Tween 80 in water (10 mL/kg) was administered orally.

### 3.4. Anxiolytic Tests

#### 3.4.1. Elevated Plus Maze (EPM) Test

The EPM was performed to evaluate the anxiolytic-like activity of CV using a previously described method [6]. The apparatus consisted of two open arms and two closed arms (50 × 10 × 40 cm^3^), made of wood. The apparatus was elevated from the surface of the floor at a height of 40 cm. The treatments administered to each group were described in Section 3.3 (Animals and Experimental Design). Each mouse was placed at the mid-point of the EPM apparatus 60 min after treatment administration. The time spent in each arm of the apparatus was recorded over a five-minute period. The test results were reported as the percentage of time spent in the open arm, as follows:

(1) $$\left(\%\right)\;\mathrm{Time}\;\mathrm{spent}\;\mathrm{on}\;\mathrm{open}\;\mathrm{arm}=\frac{\mathrm{Time}\;\mathrm{spent}\;\mathrm{on}\;\mathrm{open}\;\mathrm{arm}}{\mathrm{Time}\;\mathrm{spent}\;\mathrm{on}\;\mathrm{open}\;\mathrm{arm}+\mathrm{Time}\;\mathrm{spent}\;\mathrm{on}\;\mathrm{closed}\;\mathrm{arm}}$$

#### 3.4.2. Hole-Board Test (HBT)

The HBT apparatus was elevated at a height of 25 cm and consisted of 16 evenly distributed holes. The treatments administered to each group were described in Section 3.3 (Animals and Experimental Design). Each mouse was placed in the apparatus 30 min after treatment, and the number of times they dipped their heads in the holes was counted over a 5-min period [41].

### 3.5. Antidepressant Tests

#### 3.5.1. Forced Swim Test (FST)

The antidepressant effects of CV were assessed by the FST [42]. A transparent cylindrical glass apparatus (15 × 25 cm^2^) containing freshwater (24 ± 2 °C) at a 10 cm depth was used for this experiment. The treatments administered to each group were described in Section 3.3 (Animals and Experimental Design). Each mouse was forced to swim in the apparatus for 6 min, 60 min after treatment. The last four min were considered the immobile period, whereas the first two minutes were considered the initial adjustment period. The results were reported as the percentage of inhibition, determined as follows:

(2) $$\mathrm{Inhibition}\;\left(\%\right)\;=\frac{\mathrm{Immobile}\;\mathrm{period}\;\mathrm{of}\;\mathrm{control}\;\mathrm{group}-\mathrm{Immobile}\;\mathrm{period}\;\mathrm{of}\;\mathrm{test}\;\mathrm{group}}{\mathrm{Immobile}\;\mathrm{period}\;\mathrm{of}\;\mathrm{control}\;\mathrm{group}}$$

#### 3.5.2. Tail Suspension Test (TST)

The antidepressant activity of CV was evaluated using a method previously described by Steru et al. [43]. Each group of mice was treated as described in Section 3.3 (Animals and Experimental Design). Each mouse was suspended from the rim of the apparatus using adhesive tape placed on the tail tip (1 cm). The observations and calculations for the TST were performed as described for the FST.

### 3.6. Statistical Analysis

The differences between groups were (* *p* < 0.01 and ** *p* < 0.001) compared using one-way analysis of variance (ANOVA, followed by Dunnett’s test) in GraphPad Prism version 8.4 (GraphPad Software, San Diego, CA, USA). The results are presented as the mean ± standard error of the mean (SEM).

### 3.7. In Silico Molecular Docking Study

#### 3.7.1. Protein Preparation

Three-dimensional (3D) crystal structures of human serotonin transporter (PDB id: 5I6X) [44] and potassium channels receptor (PDB ID: 4UUJ) [45] were downloaded from the Protein Data Bank (www.rcsb.org/pdb) in pdb format. The Protein Preparation Wizard in Schrödinger-Maestro v10.1 was used to prepare and refine the structures. The other parameters were set to the system default. Minimization was performed using the force field OPLS 2005, with a root-mean-square deviation (RMSD) value of 0.30 Å.

#### 3.7.2. Ligand Preparation

For the molecular docking analysis, we selected 45 isolated compounds from *C. javanica* based on the GC–MS analysis results. The targeted compounds were downloaded from the PubChem database in sdf format. Additionally, we used paroxetine (compound CID: 43815) bound to human serotonin transporter (PDB ID: 5I6X) and diazepam (compound CID: 3016) bound to potassium channels receptor (PDB ID: 4UUJ) as positive controls for this study. The selected compounds’ three-dimensional (3D) structures were generated using Ligprep in Schrödinger suite 2015 and the OPLS_2005 force field. Using Epik 2.2 in the Schrödinger suite, we generated the ionization states of the compounds at pH 7.0 ± 2.0. For each ligand, up to 32 stereoisomers were retained.

#### 3.7.3. Receptor Grid Generation and Molecular Docking

Glide (Schrödinger Suite—Maestro version 10.1) (Schrödinger, LLC, New York, NY, USA), was used to generate receptor grids and to run molecular docking simulations [46,47]. In glide, the grids were produced using the default system and the OPLS_2005 force field. The receptor was given a cubic box of a particular size, oriented on the center of the active site residues, and a bounding box (14 Å × 14 Å × 14 Å). Glide’s standard precision (SP) scoring function was used to conduct all docking experiments, and the lowest glide score was recorded for each ligand.

### 3.8. Network Pharmacology and Pathway Analysis

The network pharmacology analysis was assessed using the STITCH platform [48,49]; multiple compound targets were analyzed by considering the 14 best-docked compounds identified from *C. javanica* leaves. Additionally, Kyoto Encyclopedia of Genes and Genomes (KEGG) analysis was utilized to identify pathways and genes involved in neuropharmacology. Moreover, the biological processes and molecular functions of these compounds were also analyzed using the STITCH platform.

## 4. Conclusions

Our findings demonstrated that in several animal models, CV had significant and dose-dependent anxiolytic and antidepressant effects, likely due to the presence of several bioactive phytoconstituents, which were identified by GC–MS analysis. The molecular docking study between the tentatively identified compounds and two receptors associated with anxiety and depression indicated good interaction scores between these two receptors and several of the identified compounds. Overall, these findings provide valuable preliminary outcomes regarding the potential anxiolytic and antidepressant activity of *C. javanica*, which required future advanced and in-depth mechanistic studies to verify the potential therapeutic applications of these bioactive compounds.

## Figures and Tables

**Table 1:** List of tentative compounds identified in a methanol extract of *Cnesmone javanica* leaves (CV) by GC–MS analysis.

SL. No.	RT (min)	Tentative Compounds	MW (amu)	PA (%)
1	3.991	Cyclopentyl acetylene	94.078	0.20
2	4.128	Hydrazine, 1,1-dimethyl-	60.069	0.23
3	4.329	Ethanethioamide	75.014	0.52
4	4.964	Phenacyl thiocyanate	177.025	0.84
5	5.204	Ethenamine, N-methyl-1-(methylthio)-2-nitro-	148.031	0.12
7	6.303	Furfural	96.021	0.65
9	9.055	2-furancarboxaldehyde, 5-methyl-	110.037	0.20
10	14.216	4H-pyran-4-one, 2,3-dihydro-3,5-dihydroxy-6-methyl-	144.042	0.26
11	14.845	5,6-epoxy-6-methyl-2-heptanone	142.099	0.14
16	18.902	2-methoxy-4-vinylphenol	150.068	0.54
17	22.742	Benzofuran, 2,3-dihydro-2-methyl-	134.073	0.16
18	25.214	2,3,5,6-tetrafluoroanisole	180.020	0.26
19	25.551 and 26.627	Megastigmatrienone	190.136	0.26 and 0.34
20	26.364	3-hydroxy-.beta.-damascone	208.146	0.20
23	29.551	Tetradecanoic acid	228.209	0.49
27	31.222	2-hexadecene, 2,6,10,14-tetramethyl-	280.313	0.25
28	31.588	1,4-eicosadiene	278.297	0.58
30	32.727	4-(2,2-dimethyl-6-methylenecyclohexyl)butanal	194.167	0.20
31	32.83	Hexadecanoic acid, methyl ester	270.256	0.88
32	33.242 and 33.665	n-hexadecanoic acid	256.240	1.17 and 5.77
33	34.254	Farnesol (E), methyl ether	236.214	0.14
35	35.462	2,6,10,14-hexadecatetraen-1-ol, 3,7,11,15-tetramethyl-, acetate	332.272	0.26
37	36.011	9,12-octadecadienoic acid, methyl ester	294.256	0.96
39	36.143	9,12,15-octadecatrienoic acid, methyl ester	292.240	1.86
40	36.372	Phytol	296.308	4.35
42	36.595	Octadecanoic acid, methyl ester	298.287	0.68
43	36.984	9,12,15-octadecatrienoic acid	278.225	10.44
44	37.356	Octadecanoic acid	284.272	1.46
45	40.503	Longipinane	206.203	0.49
46	40.646	Curan-17-oic acid, 2,16-didehydro-20-hydroxy-19-oxo-, methyl ester	354.158	0.42
47	40.88	(+)-(Z)-longipinane	206.203	0.54
48	41.241	Hexanedioic acid, bis(2-ethylhexyl) ester	370.308	7.89
49	43.598	Phthalic acid, 2-ethylhexyl tetradecyl ester	474.371	5.23
50	48.531	Squalene	410.391	6.31
51	50.219	Cyclopropanemethanol, alpha, 2-dimethyl-2-(4-methyl-3-pentenyl)-, [1.alpha.(R*),2.alpha.]-	182.167	0.34
52	50.396	2H-1-benzopyran-6-ol, 3,4-dihydro-2,8-dimethyl-2-(4,8,12-trimethyltridecyl)-, [2R-[2R*(4R*,8R*)]]-	402.350	1.30
53	50.877	2,6,10,15,19,23-hexamethyl-tetracosa-2,10,14,18,22-pentaene-6,7-diol	444.397	0.18
54	51.455	Chola-5,22-dien-3-ol, (3.beta.,22Z)-	342.292	0.30
55	51.764	β-tocopherol	416.365	0.36
56	51.981	γ-tocopherol	416.365	2.12
57	53.183 and 57.417	DL-α-tocopherol	430.381	23.53 and 3.72
58	53.457	7-(1,3-dimethylbuta-1,3-dienyl)-1,6,6-trimethyl-3,8-dioxatricyclo[5.1.0.0(2,4)]octane	234.162	0.55
59	54.373	Campesterol	400.371	0.75
60	54.825	1H-indole-2-carboxylic acid, 6-(4-ethoxyphenyl)-3-methyl-4-oxo-4,5,6,7-tetrahydro-, isopropyl ester	355.178	0.60
61	55.62	β-sitosterol	414.386	9.07
62	55.843	Androst-5,15-dien-3ol acetate	314.225	1.89

RT: retention time; MW: molecular weight; PA: peak area.

**Table 2:** Docking scores for the identified compounds from the methanol extract of *Cnesmone javanica.*

Compound Name	Docking Scores	
	4UUJ	5I6X
Cyclopentyl acetylene	−3.285	−4.557
Hydrazine, 1,1-dimethyl-	−3.474	−3.568
Ethanethioamide	−3.088	−5.163
Phenacyl thiocyanate	−3.475	−5.666
Ethenamine, N-methyl-1-(methylthio)-2-nitro-	−2.092	−2.195
Furfural	−4.077	−5.300
2-furancarboxaldehyde, 5-methyl-	−4.244	−5.998
4H-pyran-4-one, 2,3-dihydro-3,5-dihydroxy-6-methyl-	−4.887	−5.312
5,6-epoxy-6-methyl-2-heptanone	−2.828	−4.659
2-methoxy-4-vinylphenol	−3.817	−4.860
Benzofuran, 2,3-dihydro-2-methyl-	−3.491	−6.541
2,3,5,6-tetrafluoroanisole	−3.633	−6.911
Megastigmatrienone	−3.480	−5.691
3-hydroxy-beta-damascone	−3.896	−6.060
Tetradecanoic acid	+2.287	−0.427
2-hexadecene, 2,6,10,14-tetramethyl-	+0.384	−1.286
1,4-Eicosadiene	+2.442	0.337
4-(2,2-dimethyl-6-methylenecyclohexyl)butanal	−2.474	−4.984
Hexadecanoic acid, methyl ester	+1.833	−0.370
n-hexadecanoic acid	+1.793	−1.049
Farnesol (E), methyl ether	+0.176	−3.018
2,6,10,14-hexadecatetraen-1-ol, 3,7,11,15-tetramethyl-, acetate	−2.332	−6.588
9,12-octadecadienoic acid, methyl ester	+0.840	−2.495
9,12,15-octadecatrienoic acid, methyl ester	+0.046	−1.589
Phytol	−0.678	−4.012
Octadecanoic acid, methyl ester	+1.018	−1.84
9,12,15-octadecatrienoic acid	−0.123	−1.99
Octadecanoic acid	+1.519	−1.663
Longipinane	−	−5.889
Curan-17-oic acid, 2,16-didehydro-20-hydroxy-19-oxo-, methyl ester	−3.243	−5.678
Hexanedioic acid, bis(2-ethylhexyl) ester	−2.807	−5.211
Phthalic acid, 2-ethylhexyl tetradecyl ester	−1.001	−5.789
Squalene	−2.206	−5.270
Cyclopropanemethanol, .alpha.,2-dimethyl-2-(4-methyl-3-pentenyl)-, [1.alpha.(R*),2.alpha.]-	−3.075	−4.684
2H-1-benzopyran-6-ol, 3,4-dihydro-2,8-dimethyl-2-(4,8,12-trimethyltridecyl)-, [2R-[2R*(4R*,8R*)]]-	−3.741	−9.303
2,6,10,15,19,23-hexamethyl-tetracosa-2,10,14,18,22-pentaene-6,7-diol	−3.467	−6.719
Chola-5,22-dien-3-ol, (3.beta.,22Z)-	−3.054	−5.988
β-tocopherol	−3.344	−7.609
γ-tocopherol	−3.680	−8.384
DL-α-tocopherol	−4.041	−8.285
7-(1,3-dimethylbuta-1,3-dienyl)-1,6,6-trimethyl-3,8-dioxatricyclo[5.1.0.0(2,4)]octane	−	−6.085
Campesterol	−2.303	−7.242
1H-indole-2-carboxylic acid, 6-(4-ethoxyphenyl)-3-methyl-4-oxo-4,5,6,7-tetrahydro-, isopropyl ester	−3.832	−5.753
β -sitosterol	−2.626	−7.361
Androst-5,15-dien-3-ol acetate	−2.530	−5.591
**Standard (diazepam/paroxetine)**	**−3.475**	**−8.978**

**Table 3:** The docking scores of selected compounds identified in the methanol extract of *Cnesmone javanica* against potassium channels receptor (PDB ID: 4UUJ). Bold text indicates the best scores.

Compounds	Docking Score (4UUJ)	Hydrogen Bond Interactions	Hydrophobic Bond Interactions	Attractive Charges
Phenacyl thiocyanate	−3.475	Arg64	Pro63, Arg64	−
γ-tocopherol	−3.68	Asp32, Asn92	Gly30, Pro63, Trp67, Leu81	Arg64
DL-α-tocopherol	−4.041	Ser31, Arg100	Tyr62, Pro63, Arg64, Leu66, Leu81, Arg100	−
Furfural	−4.077	Lys49, Tyr104	Tyr50, Glu53, Asp102	−
2-furancarboxaldehyde, 5-methyl-	−4.244	Lys49, Tyr104	Tyr50, Asp102	−
**4H-Pyran-4-one, 2,3-dihydro-3,5-dihydroxy-6-methyl-**	**−4.887**	**Lys49, Glu53, Asp102, Tyr104**	**Lys49**	**−**
1H-indole-2-carboxylic acid, 6-(4-ethoxyphenyl)-3-methyl-4-oxo-4,5,6,7-tetrahydro-, isopropyl ester	−3.832	Asp102, Tyr104	Tyr50, Glu53, Pro63	−
2-methoxy-4-vinylphenol	−3.817	Lys49, Asp102	Tyr50, Glu53	−
Benzofuran, 2,3-dihydro-2-methyl-	−3.491	Arg64	Tyr50, Pro63, Arg64	−
2,3,5,6-tetrafluoroanisole	−3.633	Lys49, Tyr104	Lys49, Tyr50, Glu53	−
Megastigmatrienone	−3.480	Lys49, Tyr104	Tyr50, Pro63, Arg64	−
3-hydroxy-.beta.-damascone	−3.896	Lys49, Asp102	Tyr50, Pro63, Arg64	−
2H-1-benzopyran-6-ol, 3,4-dihydro-2,8-dimethyl-2-(4,8,12-trimethyltridecyl)-, [2R-[2R*(4R*,8R*)]]-	−3.741	Asp32, Asn92	Gly30, Pro63, Trp67, Leu81	Arg64
**Standard (diazepam)**	**−3.475**	**Lys49, Tyr104**	**Tyr50, Glu53, Asp102**	**−**

**Table 4:** Docking scores for selected compounds identified in the methanol extract of *Cnesmone javanica* with human serotonin receptor (PDB ID: 5I6X). Bold text indicates the best scores.

Compounds	Docking Score (5I6X)	Hydrogen Bond Interactions	Hydrophobic Bond Interactions	Attractive Charges
2-furancarboxaldehyde, 5-methyl-	−5.998	Asn177	Ala169, Ile172, Ala173, Phe341	−
Benzofuran, 2,3-dihydro-2-methyl-	−6.541	−	Ala169, Ile172,Ala173, Tyr176, Ser438, Ser439, Leu443	−
2,3,5,6-tetrafluoroanisole	−6.911	−	Ala169, Ile172,Ala173, Tyr176, Ser439, Leu443	−
3-hydroxy-.beta.-damascone	−6.060	Tyr175, Thr497	Tyr95, Ile172, Tyr176, Phe335, Phe341, Val501	−
2,6,10,14-hexadecatetraen-1-ol, 3,7,11,15-tetramethyl-, acetate	−6.588	Arg104	Tyr95, Ile172,Ala173, Tyr176, Phe335, Phe341, Val501	−
2H-1-benzopyran-6-ol, 3,4-dihydro-2,8-dimethyl-2-(4,8,12-trimethyltridecyl)-, [2R-[2R*(4R*,8R*)]]-	**−9.303**	**Ser439**	**Tyr95, Ala169, Ile172,Ala173, Tyr176, Phe335, Phe341, Val501**	**−**
2,6,10,15,19,23-hexamethyl-tetracosa-2,10,14,18,22-pentaene-6,7-diol	−6.719	Asp98, Gly100	Leu99, Trp103, Ile172, Tyr176, Ile179, Phe335, Phe341, Pro403, Val501	−
Chola-5,22-dien-3-ol, (3.beta.,22Z)-	−5.988	Glu494	Ile172, Tyr176, Phe335, Glu493	−
β-tocopherol	−7.609	Arg104	Tyr95,Arg104, Ile172, Ala173, Phe335, Phe341, Val501	−
γ-tocopherol	−8.384	Asn177	Tyr95, Ala169, Ile172,Ala173, Tyr176, Phe335, Phe341, Gly442, Leu443, Val501	−
DL-α-tocopherol	−8.285	Asn177	Tyr95, Ala169, Ile172,Ala173, Tyr176, Phe335, Phe341, Gly442, Leu443, Val501	−
Campesterol	−7.242	Arg104, Glu494	Ile172, Tyr176, Phe335, Phe341	−
β-sitosterol	−7.361	Arg104, Glu494	Ile172, Tyr176, Phe335, Phe341	−
**Paroxetine (standard)**	**−8.978**	**Tyr95, Ala96**	**Tyr95, Asp98, Ala169, Ile172, Ala173, Tyr176, Ser336, Phe341, Ser438, Ser439**	**−**

**Table 5:** Kyoto Encyclopedia of Genes and Genomes (KEGG) analysis of the compounds involved in neuropharmacology.

Pathway ID	Pathway Description	Gene Count	False Discovery Rate	Genes
4918	Thyroid hormone synthesis	5	6.98E-07	*GPX1,GPX2,GSR,PRKACA,PRKCA*
**4726**	**Serotonergic synapse**	**4**	**0.000247**	***CASP3,PRKACA,PRKCA,PTGS2***
480	Glutathione metabolism	3	0.000821	*GPX1,GPX2,GSR*
590	Arachidonic acid metabolism	3	0.00119	*GPX1,GPX2,PTGS2*
4723	Retrograde endocannabinoid signaling	3	0.00373	*PRKACA,PRKCA,PTGS2*
5146	Amoebiasis	3	0.00394	*CASP3,PRKACA,PRKCA*
5200	Pathways in cancer	4	0.00488	*CASP3,PPARG,PRKCA,PTGS2*
5206	MicroRNAs in cancer	3	0.00759	*CASP3,PRKCA,PTGS2*
4921	Oxytocin signaling pathway	3	0.00808	*PRKACA,PRKCA,PTGS2*
5016	Huntington’s disease	3	0.0121	*CASP3,GPX1,PPARG*
4913	Ovarian steroidogenesis	2	0.0155	*PRKACA,PTGS2*
4961	Endocrine and other factor-regulated calcium reabsorption	2	0.0155	*PRKACA,PRKCA*
5014	Amyotrophic lateral sclerosis (ALS)	2	0.0155	*CASP3,GPX1*
5110	Vibrio cholerae infection	2	0.0155	*PRKACA,PRKCA*
5205	Proteoglycans in cancer	3	0.0155	*CASP3,PRKACA,PRKCA*
4010	MAPK signaling pathway	3	0.0184	*CASP3,PRKACA,PRKCA*
4370	VEGF signaling pathway	2	0.0184	*PRKCA,PTGS2*
4720	Long-term potentiation	2	0.0196	*PRKACA,PRKCA*
5031	Amphetamine addiction	2	0.0198	*PRKACA,PRKCA*
4971	Gastric acid secretion	2	0.0218	*PRKACA,PRKCA*
4210	Apoptosis	2	0.0248	*CASP3,PRKACA*
4540	Gap junction	2	0.0248	*PRKACA,PRKCA*
**4727**	**GABAergic synapse**	**2**	**0.0248**	***PRKACA,PRKCA***
4911	Insulin secretion	2	0.0248	*PRKACA,PRKCA*
4912	GnRH signaling pathway	2	0.0248	*PRKACA,PRKCA*
4970	Salivary secretion	2	0.0248	*PRKACA,PRKCA*
5032	Morphine addiction	2	0.0248	*PRKACA,PRKCA*
4713	Circadian entrainment	2	0.0256	*PRKACA,PRKCA*
4750	Inflammatory mediator regulation of TRP channels	2	0.0269	*PRKACA,PRKCA*
4916	Melanogenesis	2	0.0271	*PRKACA,PRKCA*
4668	TNF signaling pathway	2	0.0314	*CASP3,PTGS2*
**4725**	**Cholinergic synapse**	**2**	**0.0314**	***PRKACA,PRKCA***
**4724**	**Glutamatergic synapse**	**2**	**0.0316**	***PRKACA,PRKCA***
4270	Vascular smooth muscle contraction	2	0.0331	*PRKACA,PRKCA*
4919	Thyroid hormone signaling pathway	2	0.0331	*PRKACA,PRKCA*
4650	Natural-killer-cell-mediated cytotoxicity	2	0.0362	*CASP3,PRKCA*
**4728**	**Dopaminergic synapse**	**2**	**0.0362**	***PRKACA,PRKCA***
**4310**	**Wnt signaling pathway**	**2**	**0.0416**	***PRKACA,PRKCA***
**5012**	**Parkinson’s disease**	**2**	**0.0418**	***CASP3,PRKACA***
5161	Hepatitis B	2	0.0418	*CASP3,PRKCA*
4261	Adrenergic signaling in cardiomyocytes	2	0.0420	*PRKACA,PRKCA*

**Table 6:** Biological processes representation of the interacting compounds.

Pathway ID	Pathway Description	Gene Count	False Discovery Rate	Genes
GO.0009743	Response to carbohydrate	5	0.00163	*CASP3,GPX1,PRKACA,PRKCA,PTGS2*
**GO.0006979**	**Response to oxidative stress**	**5**	**0.0121**	***CASP3,GPX1,GPX2,GSR,PTGS2***
GO.0009746	Response to hexose	4	0.0121	*CASP3,GPX1,PRKACA,PTGS2*
**GO.0055114**	**Oxidation-reduction process**	**7**	**0.0121**	***GPX1,GPX2,GSR,PPARG,PRKACA,PRKCA,PTGS2***
GO.1901700	Response to oxygen-containing compound	8	0.0121	*CASP3,GPX1,GPX2,GSR,PPARG,PRKACA,PRKCA,PTGS2*
**GO.0000302**	**Response to reactive oxygen species**	**4**	**0.0203**	***CASP3,GPX1,GPX2,GSR***
GO.0043627	Response to estrogen	4	0.0298	*CASP3,GPX1,PPARG,PTGS2*

Bold indicates the genes related to the neuropharmacology.

**Table 7:** Molecular function representation of the interacting compounds.

Pathway ID	Pathway Description	Gene Count	False Discovery Rate	Genes
GO.0016209	Antioxidant activity	4	0.000881	*GPX1,GPX2,GSR,PTGS2*
GO.0004601	Peroxidase activity	3	0.00499	*GPX1,GPX2,PTGS2*

## Data Availability

Available data are presented in the manuscript.
